# Anatomical Variations in Keystone Area in Nepalese Noses: A Descriptive Cross-sectional Study

**DOI:** 10.31729/jnma.8776

**Published:** 2024-10-31

**Authors:** Urmila Gurung, Kripa Dongol, Pabina Rayamajhi, Bijaya Kharel, Leison Maharjan

**Affiliations:** 1 Department of ENT-Head and Neck Surgery, Maharajgunj Medical Campus – Tribhuvan University Teaching Hospital Institute of Medicine, Maharajgunj, Kathmandu, Nepal; 2 Department of ENT, Bir Hospital, Mahaboudha, Kathmandu, Nepal; 3 Department ot ENT-Head and Neck Surgery, Patan Academy of Health Sciences, Lalitpur, Nepal

**Keywords:** *anatomical variations*, *cadaveric study*, *keystone area*, *rhinoplasty*

## Abstract

**Introduction:**

The keystone area, a crucial part of the nasal dorsum that maintains its structural stability, is known to have anatomical variations. This study aimed to evaluate these anatomical variations in Nepalese noses.

**Methods:**

This was an observational, analytical study done at a tertiary center after taking ethical approval {208(6-11)E2-077/078}. Thirty-one cadaveric noses consisting of 26 males and five females aged 24 to 70 years were dissected. The shape of the caudal margin and the overlap pattern of the nasal bone over upper lateral cartilage were assessed. The proportion of overlap was calculated. The lengths of the lateral and dorsal keystone and dorsal quadrangular cartilage were measured.

**Results:**

The most common shape of the nasal bone caudal margin was curvilinear 15 (48.39%) followed by paramedian retractions 8 (25.81%). Elongated midline was the least commonly seen in 1 (3.23%) female only. The length of the dorsal keystone ranged from 1-12 mm (mean 6.97±2.57) whilst the lateral keystone measured 1-4 mm (mean 2.22±1.20). The dorsal quadrangular cartilage measured 21-39 mm (mean 29.77±4.6). The mean overlap proportion was 23.82±7.33 (11 - 40)%.

**Conclusions:**

Anatomical variations in the structural components of the keystone area in Nepalese was seen in the study.

## INTRODUCTION

The keystone area is crucial in retaining the nasal dorsal structural integrity and aesthetic line. It has contributions from the paired nasal bones cephalically, paired upper lateral cartilages (ULCs) caudally, quadrangular cartilage (QC) anterior-inferiorly, and perpendicular plate of the ethmoid (PPE) posterior-inferiorly.^1-3^ The keystone area in the midline makes up the dorsal keystone area (DKA) and the lateral keystone area (LKA) on the lateral aspect.^4,5^ The nasal bone superimposes on the ULC by variable extent and provides strength and stability to the keystone area.^2, 3^

Disrupting the keystone area during rhinoplasty can lead to inverted V or saddle nose deformity.^4^ Since the keystone area has a thin soft tissue envelope, any change in the keystone area is evident so caution is needed during hump reduction if continuous dorsal aesthetic lines are to be achieved.^5^ Thus, it is important to know the anatomy of the keystone area, its expected dimensions, and variability to understand better its effect on the function and aesthetics of the nose and hence get the predicted outcome from nasal surgery.^2,4^

Considerable anatomical variations of the keystone area are known to occur based on racial groups and ethnicities.^2-8^ There is no publication assessing the anatomical variations of the keystone in Nepalese noses till date. Hence, this study is first of its kind that provides an anatomical baseline of the keystone area in Nepalese noses.

## METHODS

The study was an descriptive cross-sectional study conducted from February to May 2021 at Tribhuvan University Teaching Hospital, Kathmandu, Nepal after obtaining prior permission from the Department of Forensic Medicine and subsequent ethical clearance {208(6-11) E2-077/078} from the institutional review committee (IRC) of Institute of Medicine. Consent was waived by IRC as the cadavers were unclaimed bodies awaiting disposal. Cadavers with intact facial features, aged 17 or more at the time of death were included. Those with facial deformities or evidence of previous sinonasal surgical procedures or facial trauma were excluded. Thirty-one consecutive cadavers that met the inclusion criteria were assessed during this period.

All cadavers were frozen and thawed. A mid-columellar incision was made which was joined with the marginal incision in the bilateral nasal vestibule. The incision was further continued till the alar base and then extended along the alar crease. This was then continued along the nasofacial groove. The soft tissue was dissected from the entire nasal dorsum deep to the nasal superficial musculoaponeurotic system thus exposing the nasal bone, upper and lower lateral cartilage, and the quadrangular septal cartilage. The periosteum on the nasal bone was elevated and the caudal margin of the nasal bone was assessed. The caudal portion of the nasal bone was then disarticulated from the underlying upper lateral cartilage. The nasal bone was fractured with gouze and hammer and removed in piecemeal until the entire ULC overlapped by the nasal bone was exposed.

The following parameters were then assessed based on previous publications.

1. The shape of the caudal edge of the nasal bone was noted namely as curvilinear margin (curved margin without irregularity),^2^ paramedian retractions (M shaped), retracted midline (midline part retracted cephalic than the lateral caudal margin), elongated midline (midline part elongated beyond the lateral caudal margin), and combination (combination of any shapes).
2. The shape of the ULC overlapped by the nasal bone in the midline was noted namely as long midline extension (midline overlap more than 7 mm),^2^ short midline extension (midline overlap less than 7 mm) and crescent (midline and lateral part of the overlap similar in length).
3. The length of the ULC overlapped by the nasal bone was measured in the midline and laterally. The measurement in the midline represented the dorsal keystone area (DKA) and the measurement on the lateral aspect represented the lateral keystone area (LKA).
4. The length of the dorsal QC was taken as the sum of the length of DKA and the length of the QC. The length of the QC was measured in the midline from the distal edge of the nasal bone to the caudal edge of the QC.^4^
5. The proportion of QC overlapped by the nasal bone was calculated by dividing the length of DKA by the length of the whole QC.^4^

The data were entered in Microsoft® Excel (Version 16.72). SPSS (Version 23) was used for analysis of the data. Descriptive data was presented in range, mean, standard deviation, percentages.

## RESULTS

The age at the time of death of the 31 cadavers ranged from 24 to 70 years with mean age of 47.74±12.17 years. There were 26 (83.87%) male and 5 (16.13%) females.

There were 15 (48.39%) dissection in which shape of the caudal margin of the nasal bone was curvilinear paramedian retractions in 8 (25.81%). Combination was seen in 5 (16.13%) whilst retracted midline was seen in 2 (6.45%) in males. Combination included curvilinear on one side and paramedian retraction on the other side. Elongated midline was seen in 1 (3.23%) female (Table 1).

**Table 1:** Combinations of shapes of caudal edge of nasal bone with different midline overlap patterns between ULC and nasal bone (n=31)

Shape of caudal margin of the nasal bone	Midline overlap pattern between ULC and nasal bone			Total
	Long midline extension	Short midline extension	Crescent	
Curvilinear	7 (22.58)	4 (12.9)	4 (12.9)	15 (48.39)
Paramedian retractions	2 (6.45)	4 (12.9)	2 (6.45)	8 (25.81)
Elongated midline	1 (3.23)	-	-	1 (3.23)
Retracted midline	2 (6.45)	-	-	2 (6.45)
Combination	3 (9.68)	1 (3.23)	1 (3.23)	5 (16.13)
Total	15 (48.39)	9 (29.03)	7 (22.58)	31 (100)

ULC: Upper Lateral Cartilage

The length of DKA in male ranged from 3-12 mm while that of female ranged from 1-9 mm (Table 2).

**Table 2:** Measurement of different keystone area parameters (n=31).

Para meters	Gender	Range (mm)	Mean
Length of dorsal keystone area	Male (26)	3-12	7.34±2.34
	Female (5)	1-9	5±3.08
	Total (31)	1-12	6.96±2.57
Length of lateral keystone area	Male (26)	0-4	2.34±1.23
	Female (5)	1-3	1.6±0.89
	Total (31)	1-4	2.22±1.20
Dorsal length of qua drangular cartilage	Male (26)	21-39	30.80±4.12
	Female (5)	21-28	24.4±3.04
	Total (31)	21-39	29.77±4.6
Proportion of overlap between the nasal bone and qua drangular cartilage	Male (26)	11-31	23.61±6.82
	Female (5)	11-40	24.88±10.53
	Total (31)	11-40	23.82±7.33

## DISCUSSION

Weak keystone area needs to be dealt with caution during dissection and while performing osteotomies with a need for additional procedures to secure the stability of the area.^8^ Thus, a comprehensive knowledge of nasal anatomy is essential to obtain aesthetically and functionally pleasing results in rhinoplasty.^9^

Our study finding tallied partly with that of Kim et al. who also found curvilinear margin as the most common shape (44.4%) among Koreans.^2^ However, elongated midline which was the least common (3.2%) in our study, was their second commonest (22.2%). The combination margin which is attributed by the asymmetry in the paired nasal bone was encountered both in our study and that of Kim et al.^2^

Contrarily, Han et al. noted paramedian retractions in 9 (56.25%) as the commonest type in Chinese cadavers.^4^ They further divided the paramedian retractions into angular and shallow paramedian. Although less frequent, curvilinear and combination margins were also found. They claimed the distributions of these different margins to be similar to that in Korean cadavers. Knowing the shape of the caudal nasal bone margin eases and minimizes disarticulation of the nasal bone and ULC junction during osteotomy and humpectomy. ^4^

The distribution of midline overlap pattern between ULC and nasal bone in our study was similar to Korean noses.^2^ Kim et al.^2^ reported long midline extension (Co) in 9 (50%), short midline extension (Bo) in 4 (22.2%) and crescent shaped (Ao) in 3 (16.6%). In addition, a further combination of types Ao+Co and Bo+Co in 2/18 (11.1%) were also noted. The authors thus highlighted the presence of a lot of variability in the shape of the overlap between ULC and the nasal bone. Similar variability has been reported in adult Caucasian cadavers also.^5^

The length of the overlap between ULC and nasal bone is synonymous to the length of DKA in the midline and LKA laterally. The overlap tapers in the lateral part indicating the LKA to be shorter than the DKA.^10^

The length of the DKA in our study was similar however the LKA was less than that of the Koreans, Chinese, and Caucasians. Kim et al.^2^ reported a mean length of 7 mm (range 4-10 mm) in the midline and 3.1 mm (range 0-7 mm) in the paramedian area. Similar measurement of 7±2 mm overlap was observed in the study by the same group based on magnetic resonance imaging. In the Chinese cadavers, Han et al.^4^ found the mean length to be 6.47±2.50 mm in the midline, 3.53±2.23mm on the left, and 3.81±2.56mm on the right. The mean DKA length amongst Caucasians was 6.5 mm (range 0.5-11.5 mm) in the study by Simon et al.^3^ There was no statistical difference in their measurements of the midline overlap when compared with the data set from study by Kim et al.^2^ This finding was interestingly contradictory more so because anthropometric differences between the Caucasian and the Asian noses are known to exist. Other studies however found longer DKA in Caucasians.^5,10^

There were observed differences even in the same race when different methodologies were used. The length of the DKA obtained in the cadaveric dissection i.e. a mean of 7 mm as per Kim et al.^2^ differed from the CT scan studies i.e. 9.8±3.3 mm and 9.2±2.4 mm for male and female patients, respectively (p=0.13) as noted by Kim & Jang^8^ in Koreans. Based on the sagittal images on CT scan of the paranasal sinuses of 177 Japanese patients, Nomura et al.^11^ found the overlap ranging from 0-15.9 mm with a mean of 8 mm. There was no difference between males and females however, it decreased with age. So, this study found the length of overlap in Japanese longer than that of the Koreans and Chinese but shorter than that of the _Caucasians._^5,10,12,13^

In our study, males had statistically significantly longer DKA and LKA overlap as compared to females. Surprisingly Carr et al.^12^ found longer overlap in females than in males. However, other studies found no difference based on gender.^3,8,11,13^

There is a lot of disparity between age and the length of the overlap. Some studies found shorter overlap in older specimens, possibly due to septal cartilage calcification with aging^2,11^ whilst others found no difference in the overlap length based on age.^3,13^ The relation between age and overlap length was not assessed in this study.

The strength of the keystone area depends on the area of contact between the ULC and the nasal bone. This differs based on the type of assembly between the caudal edge of nasal bone and the midline overlap patterns.^2^ Retracted midline caudal edge combined with crescent overlap pattern forms the weakest assembly whereas elongated midline caudal edge with long midline extension overlap forms the strongest assembly. In our study, the most common assembly was the curvilinear caudal margin of nasal bone with a long midline extension of the overlap, providing moderate strength to the keystone area. Hence, in Nepalese noses, although the risk of disrupting the keystone area is not alarming, caution should still be taken during hump removal.

These variations in the overlap pattern could be the reason for the different outcomes in different patients as saddle nose deformity does not always occur in all patients after nasal procedures like hump resection and osteotomies.^2^ Asians who generally tend to have shorter, wider, and less-projecting noses as compared to Caucasians^7^ have shorter nasal bones and cartilages.^2,14^ Carr et al.^12^ described nasal bones as being short if measuring less than a third of the dorsal length. In such conditions, caution should be taken to avoid disrupting the chondroethmoid junction with.

Our results were slightly more than that observed in Chinese cadavers by Han et al.^4^ The length of the whole dorsal QC was 20 to 33 mm (25.63±4.27 mm). Comparing their mean to Simon's Caucasian measurement of 27.1±4.30 mm (20.0-34.5 mm) there was no statistically significant difference. MRI study in Koreans found the length of the cartilaginous dorsum to be 26±4 mm with no significant difference between genders.^2^ However, in the CT scan study in Koreans, the mean length of the cartilaginous dorsum was 28.5±2.7 mm for male patients and 25.5±2.1 mm for female patients (p-0.01) suggesting males have significantly longer dorsal cartilage than females.^8^ Godley found the length of the Caucasian cartilaginous dorsum comparatively shorter (21.5 mm, even shorter in females by 5.4 mm on average) than the Korean and Chinese measurements by Kim et al. and by Han et al. respectively.^6,2,4,^

Simon et al.^3^ and Han et al.^4^ found a similar proportion of QC overlapped by the nasal bone amounting to 23.8% and 25±8% respectively which was comparable to this study (23.82%). So, only about 24% of the dorsal septum is securely fixed at the keystone area whilst the rest of the dorsal septum relies on the loose support from the ULC and LLC. Hence, this area can become unstable even with minor changes in the overlap.

The strength of this study includes it being the first to assess the keystone area in cadavers of Nepalese origin, hence it forms a baseline for future studies. The measurements were taken by the principal investigator to avoid interobserver bias. Limitations of this study include small sample size, disparity in the gender distribution of the cadavers, and exclusion of ethnic variations. A study with a larger sample size with an equal gender distribution and assessment based on ethnicity can give more precise information on the prevalence of the anatomical variations of the keystone area in Nepalese.

## CONCLUSIONS

Anatomical variations in the structural components of the keystone area in Nepalese noses were evident. Such variance could probably be secondary to ethnic structural differences in facial features. Awareness of such variability is of utmost importance whilst planning and performing rhinoplasty.
